# Targeting *KRAS* Mutant Non-Small-Cell Lung Cancer: Past, Present and Future

**DOI:** 10.3390/ijms21124325

**Published:** 2020-06-17

**Authors:** Iris Z. Uras, Herwig P. Moll, Emilio Casanova

**Affiliations:** 1 Department of Pharmacology, Center of Physiology and Pharmacology & Comprehensive Cancer Center (CCC), Medical University of Vienna, 1090 Vienna, Austria; 2 Department of Physiology, Center of Physiology and Pharmacology & Comprehensive Cancer Center (CCC), Medical University of Vienna, 1090 Vienna, Austria; herwig.moll@meduniwien.ac.at (H.P.M.); emilio.casanova@meduniwien.ac.at (E.C.); 3 Ludwig Boltzmann Institute for Cancer Research (LBI-CR), 1090 Vienna, Austria

**Keywords:** lung adenocarcinoma, NSCLC, KRAS, targeted therapy, immunotherapy, metabolic rewiring, p53, STK11, EGFR, degraders

## Abstract

Lung cancer is the most frequent cancer with an aggressive clinical course and high mortality rates. Most cases are diagnosed at advanced stages when treatment options are limited and the efficacy of chemotherapy is poor. The disease has a complex and heterogeneous background with non-small-cell lung cancer (NSCLC) accounting for 85% of patients and lung adenocarcinoma being the most common histological subtype. Almost 30% of adenocarcinomas of the lung are driven by an activating *Kirsten rat sarcoma viral oncogene homolog* (*KRAS*) mutation. The ability to inhibit the oncogenic *KRAS* has been the holy grail of cancer research and the search for inhibitors is immensely ongoing as *KRAS*-mutated tumors are among the most aggressive and refractory to treatment. Therapeutic strategies tailored for *KRAS^+^* NSCLC rely on the blockage of KRAS functional output, cellular dependencies, metabolic features, KRAS membrane associations, direct targeting of *KRAS* and immunotherapy. In this review, we provide an update on the most recent advances in anti-KRAS therapy for lung tumors with mechanistic insights into biological diversity and potential clinical implications.

## 1. Introduction

Lung cancer remains the leading cause of cancer-related lethality worldwide, with nearly 1.6 million deaths, and the five-year survival rate is still below 20% [1]. Outcomes with platinum-based chemotherapy as first-line treatment in patients with stage IV non-small-cell lung cancer (NSCLC) are poor, with a clear need for improved treatments and for individualized therapeutic approaches for each patient [2,3,4]. NSCLC molecular profiling is a key factor in therapy decision making, with a number of emerging oncogenic targets and active targeted agents. Somatic mutations in *epidermal growth factor receptor* (*EGFR*) and rearrangements in *anaplastic lymphoma receptor tyrosine kinase* (*ALK*), *proto-oncogene tyrosine-protein kinase ROS* (*ROS*) and *RET proto-oncogene* (*RET*) have been nominated as solid predictive biomarkers and attractive drug targets in NSCLC [5,6]. The *rat sarcoma proto-oncogene (RAS)* family, the most frequently mutated oncogene family in cancer, however, has been defined as undruggable [7] and, despite four decades of efforts, no potent anti-RAS therapy is currently used in routine clinical practice.

**Figure 1:**
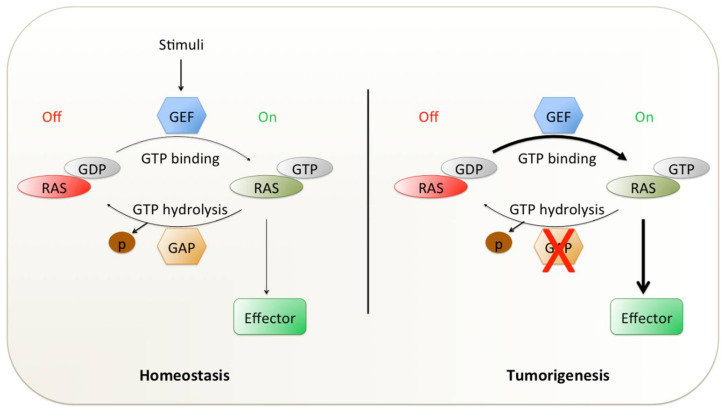
The two states of RAS family GTPases. GTP- and GDP-bound states are directed by GEFs and GAPs. GEFs stimulate the exchange of GDP to GTP, promoting activation of RAS (On). GAPs drive GTP hydrolysis and return to inactive GDP-bound status (Off). The thickness of the arrows indicates the strength of GTP binding as well as the (hyper)activation of downstream effectors. The red cross indicates the impairment of GTP hydrolysis. GEF: guanine nucleotide exchange factor; GAP: GTPase-activating protein; GTP: guanosine triphosphate; GDP: guanosine diphosphate; RAS: rat sarcoma proto-oncogene

**Figure 2:**
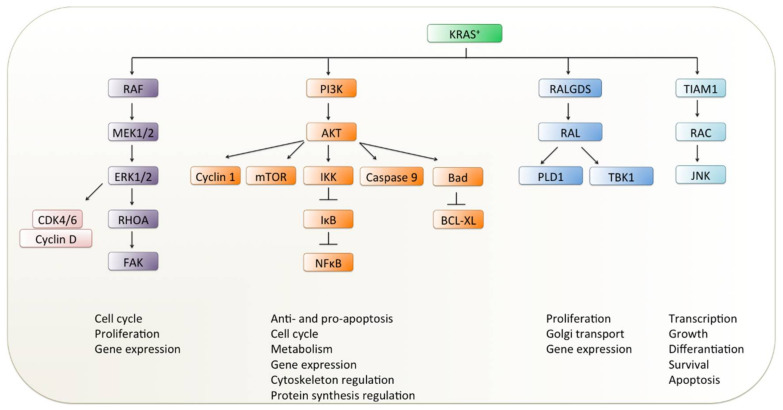
KRAS signaling in non-small-cell lung cancer (NSCLC). Schematic presentation of signaling pathways initiated by oncogenic *KRAS* aberrations is illustrated in a simplified fashion. RAF: rat fibrosarcoma; MEK: mitogen-activated protein kinase kinase; ERK: extracellular regulated kinase; PI3K: phosphoinositide 3-kinase; AKT: protein kinase B; mTOR: mechanistic target of rapamycin kinase; RALGDS: Ral guanine nucleotide dissociation stimulator; KRAS: Kirsten rat sarcoma viral oncogene homolog; CDK4/6: cyclin-dependent kinase 4/6; RHOA: Ras homolog family member; FAK: focal adhesion kinase; IKK: IkappaB kinase; IκB: nuclear factor of kappa light polypeptide gene enhancer in B-cells inhibitor; NF-κB: nuclear factor ‘kappa-light-chain-enhancer’ of activated B-cells; Bad: BCL2-associated agonist of cell death; BCL-XL: B-cell lymphoma-extra large; RAL: Ras-like protein; PLD1: phospholipase D1; TBK1: TANK binding kinase 1; TIAM1: T lymphoma invasion and metastasis-inducing protein 1; RAC: Ras-related C3 botulinum toxin substrate 1; JNK: c-Jun N-terminal kinase.

The *RAS* gene family members encode a membrane-bound small GTPase and switch between the active guanosine triphosphate (GTP)-bound and inactive guanosine diphosphate (GDP)-bound state [8] (Figure 1). The activation of RAS signaling is tightly controlled by the regulator factors that promote the GDP–GTP exchange (guanine nucleotide exchange factors; GEFs) or affect its GTPase activity (GTPase-activating proteins; GAPs). GEFs and GAPs bind to one or both of the binding pockets on RAS (known as switch I and switch II regions) [9,10]. This process links upstream cell surface receptors such as EGFR, fibroblast growth factor receptor (FGFR) and human epidermal growth factor receptors 2–4 (HER2–4/ERBB2–4) to downstream pathways (e.g., rat fibrosarcoma/mitogen-activated protein kinase kinase/extracellular regulated kinase (RAF/MEK/ERK), phosphoinositide 3-kinase/protein kinase B/mechanistic target of rapamycin kinase (PI3K/AKT/mTOR) and Ral guanine nucleotide dissociation stimulator (RALGDS-RA)) promoting cell proliferation, differentiation or cell death [11,12,13,14,15,16] (Figure 2). Oncogenic mutations in RAS proteins impair their ability for GTP hydrolysis, resulting in the accumulation of GTP-bound active RAS (Figure 1) and hyperactivation of downstream signaling cascades that lead to uncontrolled cell proliferation and survival.

The three RAS family members that have been extensively evaluated in humans are Kirsten rat sarcoma viral oncogene homolog (KRAS), neuroblastoma rat sarcoma viral oncogene homolog (NRAS) and Harvey rat sarcoma viral oncogene homolog (HRAS). The frequency and distribution of *RAS* mutations are not uniform. KRAS is the isoform most frequently altered in 86% of *RAS* mutant cancer cases, followed by NRAS 11% and HRAS 3% [17]. *KRAS* aberrations are mainly found in lung, pancreatic and colon cancer, *NRAS* in melanoma and *HRAS* in bladder and head and neck squamous cancers [18]. *KRAS* mutations account for approximately 30% of lung adenocarcinomas in Western countries and for 10–15% of cases in Asia [19]. The *KRAS^G12C^* mutation is highly prevalent in patients suffering from lung adenocarcinoma (13% of total lung adenocarcinoma) and account for >50% of all *KRAS* mutant cases [20].

**Figure 3:**
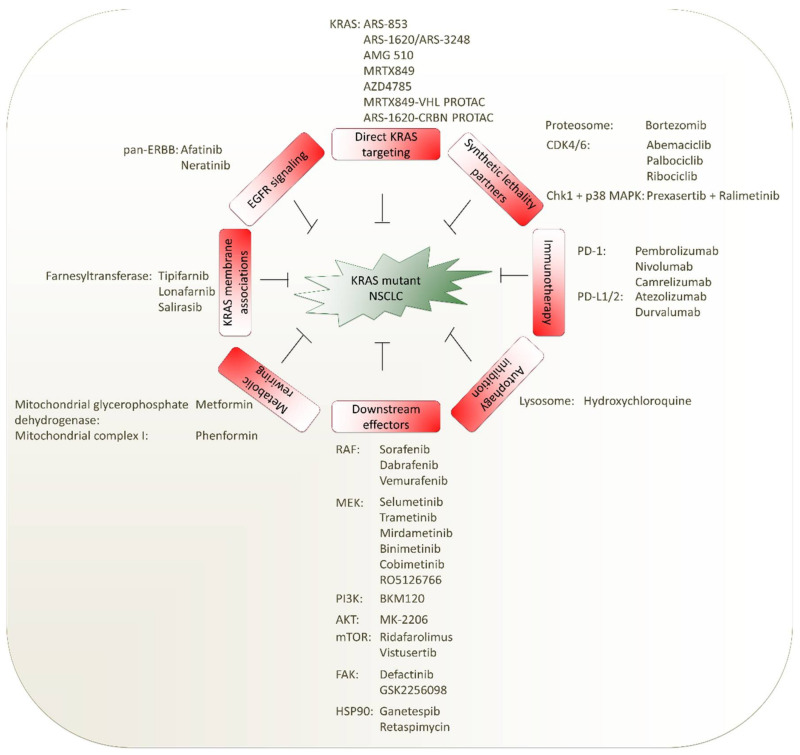
Therapeutic approaches to tackle *KRAS* mutant NSCLC in patients. Selected compounds and their targets are listed. CDK4/6: cyclin-dependent kinase 4/6; Chk1: checkpoint kinase 1; CRBN: cereblon; PROTAC: proteolysis-targeting chimeras; VHL: von Hippel–Lindau disease tumor suppressor; HSP90: heat shock protein 90; RAF: rat fibrosarcoma; MEK: mitogen-activated protein kinase kinase; PI3K: phosphoinositide 3-kinase; AKT: protein kinase B; mTOR: mechanistic target of rapamycin kinase; KRAS: Kirsten rat sarcoma viral oncogene homolog; FAK: focal adhesion kinase; ERBB: human epidermal growth factor receptor; PD-1: programmed cell death protein 1; PD-L: programmed cell death 1 ligand; MAPK: mitogen-activated protein kinase.

Although KRAS was one of the earliest oncogenic drivers discovered, effective KRAS-targeted therapies still remain elusive. *KRAS* mutant lung cancers have worse outcomes in both early stage and advanced metastatic settings, illustrating the critical need for novel agents targeting *KRAS*-driven NSCLC. Numerous therapeutic strategies have been developed including targeting KRAS membrane associations, synthetic lethality partners, blockage of downstream signaling cascades, targeting metabolic reprogramming, direct targeting of *KRAS* and immunotherapy (Figure 3). In this review, we provide a critical assessment of past efforts and discuss the most promising improvements for future success.

## 2. Molecular Diversity in *KRAS* Mutant NSCLC Influences Effective Targeting

*KRAS*-mutated NSCLC is composed of a heterogeneous set of distinct diseases. Co-occurring genetic events, distinct *KRAS* mutation subtypes, and mutant *KRAS* allelic content are among the main drivers that contribute to direct clinical implications.

*KRAS* mutant NSCLC often appears with additional genetic alterations—a feature not shared by other oncogene-driven NSCLC. Recent RNA sequencing efforts in *KRAS^+^* NSCLC defined three expression clusters dominated by the co-mutations in serine/threonine kinase 11 (*STK11,* also known as *liver kinase B1 (LKB1))* and *tumor protein p53 (p53)*, inactivation of *cyclin-dependent kinase inhibitor 2A/B* (*CDKN2A/B*) and low levels of *thyroid transcription factor-1* [21]. *KRAS/STK11* driven tumors were characterized by *hypoxia-inducible factor-1 alpha*-mediated metabolic reprogramming and adaptation to oxidative and endoplasmic reticulum stress and showed enrichment in *Kelch-like ECH-associated protein 1* (KEAP1) mutations^21^. *KRAS/CDKN2A/B*-mediated tumors were enriched in gene expression signatures of gastrointestinal neoplastic processes and wildtype p53 transcriptional activity [21]. The presence of major co-mutations is of prognostic significance (e.g., *STK11* or *KEAP1* mutations are associated with worse overall survival) and of predictive value for therapeutic vulnerabilities [21,22,23].

Mutational subtype of individual *KRAS* mutations is of greater relevance than the categorical evaluation of its presence or absence. To date, the most frequent *KRAS* mutations in NSCLC have been documented at codons 12 and 13 [24]. Among those, glycine 12 to cysteine (G12C) and glycine 12 to valine (G12V) are the most common subtypes associated with a smoking history, whereas glycine 12 to aspartic acid (G12D) is mainly found in never smokers [24,25,26]. Each amino acid substitution differs in its binding affinity for downstream effectors: *KRAS*^G12D^ had higher affinity for PI3K/AKT signaling, whereas *KRAS^G12C^* or *KRAS^G12V^* led to low levels of phosphorylated AKT and increased RAL activation compared with other mutations or wildtype situation [27]. Two independent studies in metastatic patients and in those with surgically resected NSCLC are in favor with the idea that different codon variants may promote activation of distinct transcriptional networks which impact on prognosis and/or therapeutic susceptibilities: *KRAS^G12C^* or *KRAS^G12V^* positivity was associated with worse disease-free and overall survival when compared with other *KRAS* variants or wildtype protein, at least partly due to increased levels of epithelial to mesenchymal transition genes and lower levels of genes predicting KRAS dependency [28,29]. However, reports in adjuvant and advanced settings failed to prove any prognostic significance based on *KRAS* mutational subtype [21,30,31].

A common feature among *KRAS* mutant NSCLC is the metabolic rewiring of tumors towards anabolism [32]. Not only does the presence of certain co-mutations define distinct metabolic and redox management profiles [21], but also the mutant *KRAS* allelic content is a major contributor [33]. *KRAS^G12D/G12D^* cells exhibited a glycolytic switch towards glutathione biosynthesis and a higher metastatic potential. These changes were recapitulated in spontaneous advanced murine lung tumors with a high frequency of *KRAS^G12D^* copy gain (homozygous) but not in the corresponding early tumors (*KRAS^G12D^* heterozygous). Tumors with *KRAS^G12D^* allelic enrichment had lower survival rates than heterozygous tumors and were highly susceptible to the glycolytic inhibitor 2-deoxyglucose and the glutathione synthesis inhibitor buthionine sulfoximine. These data suggest that *KRAS* mutant NSCLC may be classified on a metabolic basis for a better therapeutic outcome.

Taken together, one common feature of earlier studies without succeeding to improve outcome upon treatments was that detailed information on the genetic context of individual tumors was unobtainable in the vast majority of patients [34]. Hence, future efforts of anti-RAS therapy in NSCLC should be stratified based on the primary drivers of its biological diversity.

## 3. Treatment Failures of the Past in Mutant *KRAS* NSCLC

For almost four decades, a therapeutic breakthrough in targeting *KRAS* has been hampered by structural and biochemical obstacles, prompting the widely held perception that RAS proteins are untargetable. Several factors contribute to these obstacles: (i) KRAS binds to GTP with picomolar affinity in its active state and this interaction needs more potent inhibition than that granted by traditional blockers; and (ii) clear druggable pockets on KRAS large enough for small-molecule binding were/are missing [7,34]. Therefore, tackling of mutant *KRAS* has been focused on inhibition of its membrane association/subcellular localization, identification of synthetic lethality partners, and inhibition of downstream effectors. Yet neither strategy has yielded significant successes for many years.

### 3.1. Blocking KRAS Membrane Association

RAS proteins require membrane associations for their biological activation, making this association a logical target for anti-RAS drug development. Once synthesized in the cytosol, they become lipophilic for membrane attachment. Lipid alteration of RAS proteins by the linkage of 15-carbon farnesyl polyisoprene onto the cysteine of C-terminal CAAX motifs is an obligate step in this process catalyzed by the farnesyltransferase (prenylation) [35]. Farnesyltransferase inhibitors (FTIs) represent the most well-known example of treatment failure in targeting mutant *RAS*. The first-generation drugs tipifarnib and lonafarnib were well tolerated but with poor clinical efficacy in advanced NSCLC [36,37]. Such lack of activity was thought to be due to the alternative prenylation of RAS by geranylgeranyltransferase I (GGTase I) [38,39]: the geranylgeranylation of KRAS still permits its membrane localization and signal transduction, overcoming the effect of FTI. However, dual inhibition of farnesyltransferase and geranylgeranyltransferase did not yield any clinical benefit [40]. The second-generation FTI salirasib, at the dose and schedule applied in a phase II trial, exhibited insufficient activity in the treatment of *KRAS* mutant lung adenocarcinoma [41] despite the promising preclinical data [42,43]. Such lack of efficacy might be due to alternative resistance mechanisms (e.g., *KRAS* gene amplification) or off-target effects and demands a better understanding. Of note, considering *RAS* mutant isoforms in these settings has proven essential for designing and delivering trials: contrary to KRAS and NRAS, HRAS protein does not undergo alternative prenylation and application of FTIs to *HRAS* mutant cell lines has been effective [39].

Targeting of enzymes involved in post-prenylation of RAS such as RAS-converting enzyme 1 (RCE1) and isoprenyl carboxyl methyltransferase (ICMT1) renewed interest in efforts for anti-RAS therapeutics. Although *Rce1* or *Icmt1* deficiency in mouse embryonic fibroblasts impaired *HRAS*-mediated transformation, later studies provided puzzling results about loss of these enzymes: (i) absence of *Rce1* in mouse hematopoietic cells accelerated *KRAS^G12D^*-driven myeloproliferative disease [44], whereas inactivation of *Icmt1* in the same mouse model had a protective effect; [45] (ii) *Icmt1* deficiency in the pancreas enhanced *KRAS^G12D^*-driven neoplastic progression [46]. These discrepancies might be explained by the fact that both enzymes have up to 300 additional substrates that may be differentially sensitive to enzyme blockage in a context-dependent manner. Several RCE1 and ICMT1 inhibitors have been developed and characterized [47,48,49] but further optimization is needed before their entrance in human trials.

Other enzymatically regulated modifications involved in RAS trafficking, subcellular localization and/or effector interactions that are promising directions to achieve the goal of disrupting RAS include de-/palmitoylation, phosphorylation, endothelial nitric oxide synthase-catalyzed nitrosylation, ubiquitination and acetylation. However, developing potent and selective inhibitors might be challenging due to a wide spectrum of substrates. The precise biological mechanisms driving KRAS membrane association, the enzymology of such modifications, the significance of their stoichiometry and their potential therapeutic value should be fully characterized before entering clinical testing [7,35].

### 3.2. Synthetic Lethality Partners as Therapeutic Vulnerabilities

One alternative indirect approach to attack KRAS function in cancer has been to seek targets that are essential in cells bearing activated *KRAS* oncogene but not those without. This so-called synthetic lethal approach in cancer treatment, rooted in ideas from invertebrate genetics [50,51], has been inspired most strongly by the use of poly(ADP-ribose)polymerase (PARP) inhibitors in the clinic to treat *breast cancer gene (BRCA)*-negative tumors [52,53].

Several drug library screens or cell line-based genetic screens using RNA interference (RNAi)-mediated expression silencing or clustered regularly interspaced short palindromic repeats/CRISPR-associated protein 9 (CRISPR/Cas9) technology have been carried out to identify genes that are uniquely essential to *KRAS* mutant but not wildtype cells. Their results can be categorized into the following cellular processes: cell cycle and mitosis (survivin, targeting protein for Xklp2 (TPX2), polo-like kinase 1 (PLK1), anaphase-promoting complex/cyclosome (APC/C), cell survival (Wilms tumor 1 (WT1), B-cell lymphoma-extra large (BCL-XL1), transcriptional programs (GATA-binding protein 2 (GATA2), snail family transcriptional repressor 2 (SNAIL2) and growth and survival signals (TANK binding kinase 1 (TBK1), mitogen-activated protein kinase kinase kinase 7 (MAP3K7/TAK1) [7]. The findings of KRAS dependency on kinases (e.g., TBK1 [54]) and transcription factors (e.g., GATA2 [55,56]) suggest the involvement of additional cellular pathways to support the viability of *KRAS* transformed cells. Unfortunately, these large-scale screens suffer from various limitations including high genetic complexity of *KRAS* mutant cells (large number of activating co-occurring alterations), experimental artefacts (e.g., choice of cell line, inconsistency of methodology), low magnitude of synthetic lethality effect and lack of validation [57,58]. The problematic can be exemplified as follows: (i) after its identification as a synthetic lethal partner using an RNAi screen in *KRAS* mutant cell lines [59], inhibition of STK33′s kinase activity by the small molecule BRD-8899 proved ineffective [60]; (ii) although three independent groups defined *GATA2* as essential for survival of *KRAS* mutant NSCLC [55,56,61], Tessema et al. showed that *GATA2* is epigenetically repressed in human and mouse lung tumors and its inhibition is not a valid therapeutic strategy for *KRAS* mutant lung cancer [62]. In fact, the screens have been notable for little or no target overlap between their results, with the exception of proteosome-related genes [58]. Bortezomib, a proteosome inhibitor, was associated with modest anti-tumor activity and durable disease control in a small fraction of patients with *KRAS*^G12D^ mutant lung tumors [63,64]. However, *KRAS*^G12D^ mutation alone is not a robust predictor of response and further evaluation should only be performed after elucidation of co-mutations and histologic subtype that may predict therapy sensitivity [63,64]. It is also likely that significant response would be achieved when combined with other agents. Apart from chemotherapeutics, a possible combination partner could be MEK inhibition, currently the only targeted therapy with selectivity for *KRAS* mutations in the clinic (approved for *B-RAF^V600E^*-mutated NSCLC). In fact, the essentiality of MEK signaling to *RAS*-mediated transformation has led to a combinatorial synthetic lethality screen with MEK inhibitors, which has revealed the anti-apoptotic BH3 family gene BCL-XL as a potential sensitivity hit [65].

Another interesting synthetic lethal strategy describes combined inhibition of discoidin domain receptor 1 (DDR1) and NOTCH signaling [66]. The tyrosine kinase receptor DDR1 was identified as the top-scoring gene in transcriptional profiling of *KRAS^G12V^*-driven mouse hyperplasias. The concomitant blockage of DDR1 and NOTCH signaling induced regression of *KRAS/p53* co-mutated patient-derived xenografts (PDX) with a therapeutic efficacy greater than standard chemotherapy.

A further potential therapeutic application in NSCLC has been unveiled by recent mouse work: genetic and chemical blockage of cyclin-dependent kinase 4 (CDK4) was synthetic lethal in genetically modified *KRAS*-driven lung tumor models [67]. Consistently, a multi-tumor phase I trial reported that the CDK inhibitor abemaciclib (Verzenios, Lily; with greater selectivity to CDK4 than its close homolog CDK6) achieved a higher disease control rate in NSCLC patients with *KRAS* mutations than without [68]. However, in a phase III trial comparing abemaciclib vs. erlotinib (an EGFR inhibitor) as second- or third-line treatment of advanced *KRAS* mutant NSCLC (NCT0215631), overall survival (the primary endpoint) did not differ between the two study arms [69]. Palbociclib (Ibrance, Pfizer), another U.S. Food and Drug Administration (FDA)-approved CDK4/6 inhibitor, is in clinical evaluation for *KRAS* mutant NSCLC either in combination with MEK inhibitors (NCT02022982, NCT03170206) or with the glutaminase inhibitor telaglenastat (CB-839) (NCT03965845). Another trial is underway to assess the preliminary efficacy of the CDK4/6 inhibitor ribociclib (Kisqali, Novartis) in combination with protein tyrosine phosphatase non-receptor type 11 (PTPN11) inhibitor TNO155 (NCT04000529). Further cell cycle-related proteins have been described as synthetic lethal partners of KRAS signaling in vitro and in vivo: mitotic catastrophe was observed in *KRAS*-mutated but not wildtype tumors when exposed to inhibitors of checkpoint kinase 1 (Chk1) and MAPK-activated protein kinase 2 (MK2) [70]. A phase I trial (NCT02860780) has been conducted to evaluate the combination of prexasertib (a Chk1 inhibitor) and ralimetinib (a p38 MAPK inhibitor) in patients with advanced or metastatic cancer including NSCLC with results being not available as yet.

Despite recent advances, to date, KRAS synthetic lethality has fallen short of its original promise and remains unproven as routine clinical practice to attack *KRAS^+^* NSCLC.

### 3.3. Targeting Downstream Effectors of KRAS Signaling

Many of the downstream effectors of the KRAS pathway (e.g., RAF, MEK, PI3K and mTOR) are rational therapeutic targets and their potential value has been evaluated in many NSCLC clinical trials. Different *KRAS* codon subtypes show a preference for activating different downstream signaling [27]; hyperactivation of the MAPK pathway is a key feature in *KRAS*-driven lung cancer. One explanation for this is that the most common subtype G12C exhibits more prominent engagement with MAPK signaling [71]. Several clinical trials are underway evaluating the potential of the inhibition of KRAS functional output by single agents or in combinatorial schedules with chemotherapy, other targeted agents and/or radiation therapy (e.g., NCT03415126, NCT01859026, NCT02964689, NCT03704688, NCT01306045, NCT01912625, NCT02185690, NCT04121286, NCT03990077, NCT02450656, NCT03989115, NCT04045496).

#### 3.3.1. RAF Inhibitors

The direct RAS downstream effectors within the RAF/MEK/ERK pathway are the RAF kinases v-Raf murine sarcoma 3611 viral oncogene homolog 1 (A-), v-Raf murine sarcoma viral oncogene homolog B (B-) and v-Raf-1 murine leukemia viral oncogene-like protein 1 (C-RAF). One of the first drugs aimed at blocking KRAS/RAF/MEK/ERK signaling was sorafenib [72]. The multikinase inhibitor sorafenib exerts modest activity against RAF kinases yet efficacy in patients with *KRAS* mutant lung tumors is rather disappointing with an increase of 3 months in median progression-free survival (PFS) [73,74,75,76]. Further, *KRAS* mutational status was not of any predictive value in the BATTLE and MISSION trials when sorafenib was administered as a single agent in relapsed/refractory NSCLC patients [73,75,76].

Dabrafenib and vemurafenib, both type I B-RAF inhibitors, are potent in targeting *B-RAF* mutant NSCLC but show no benefit in patients with *KRAS* mutations. This is explained by the fact that C-RAF but not B-RAF plays a unique role in mediating KRAS oncogenic signaling. Ablation of *C-RAF* recapitulated the effects of disrupting *KRAS* and completely blocked tumor initiation without any significant toxicities observed in *KRAS*-driven lung tumor mouse models [77,78]. In advanced tumors driven by *KRAS^G12V^/p53*, systemic abrogation of C-RAF expression induced regression due to massive apoptosis by a mechanism independent of canonical MAPK signaling which is also essential for normal homeostasis^79^. The observed therapeutic benefit was likely due to the disrupted interaction between C-RAF protein and other partners (e.g., B-cell lymphoma 2 (BCL2), apoptosis signal-regulating kinase 1 (ASK1), serine/threonine kinase 3 (STK3), and Rho-associated coiled-coil containing protein kinase 2 (ROCK2)) [79]. This might explain, at least partly why *C-RAF* absence did not induce any significant adverse events, in contrast to MEK inhibitors. The catalytic activity of C-RAF is not required for the *KRAS*-mediated transformation [79]: three C-RAF inhibitors (MLN2480, GW5074, and PLX8394) designed to block the kinase activity but not the protein expression failed to yield any therapeutic effect [71,79]. Opposing the currently ongoing efforts to generate C-RAF kinase inhibitors, these studies rather encourage the development and implication of C-RAF specific degraders [72,80].

#### 3.3.2. MEK Inhibitors

Genetic studies have helped to dissect the requirements for each member of the MAPK pathway in *KRAS* induced tumors. Individual MEK1/2 kinases are dispensable for the initiation of lung adenocarcinomas driven by *KRAS^G12V^*, whereas complete elimination of MEK expression efficiently prevents tumor development [77]. Several MEK inhibitors have been developed—most of which are allosteric kinase inhibitors. They are highly specific and less likely to cause off-target toxicity due to their affinity for a unique allosteric pocket on MEK, rather than for the catalytic site with its higher homology to other kinases [81]. However, MEK inhibition is notably susceptible to adaptive feedback via reactivation of MAPK signaling [82].

A phase II trial failed to show improvement in objective response rate (ORR) or PFS upon concomitant combination therapy of selumetinib (an allosteric MEK inhibitor) and erlotinib (an anti-EGFR inhibitor) over monotherapy in *KRAS* mutant and *KRAS* wildtype advanced NSCLC [83]. The ORR in the *KRAS* mutant arm was 0% for selumetinib alone and 10% for the combination. Combination therapy resulted in increased toxicities, requiring dose reductions and discontinuation. Programmed cell death 1 (PD-1) expression on regulatory T cells (Tregs), T cell immunoglobulin mucin-3 (Tim-3) on CD8^+^ T cells and T helper 17 (Th17) levels were associated with PFS and overall survival in patients receiving selumetinib [83]. Further, in an unselected NSCLC population, there was no suggestion that selumetinib monotherapy demonstrates superiority with standard second-line pemetrexed-based chemotherapy [84]. In a randomized phase II trial, concomitant treatment with selumetinib plus docetaxel (a taxane chemotherapy) suggested an incremental survival advantage but with higher numbers of adverse events than with docetaxel alone in previously treated *KRAS* mutant NSCLC [85]. However, these results could not be recapitulated in the larger phase III SELECT-1 trial (NCT01933932) [86]. Such lack of therapeutic response might be caused by the impact of co-existing genetic modifiers. In a preclinical study using mouse models, simultaneous loss of either *p53* or *STK11*, both clinically relevant tumor suppressors, markedly impaired the response of *KRAS* mutant tumors to docetaxel alone [87]. The addition of selumetinib offered substantial benefit for mice bearing *KRAS* and *KRAS/p53* mutations, whereas *KRAS/STK11* mutant mice displayed primary resistant to the therapy due to activation of parallel signaling pathways such as AKT and v-Src avian sarcoma (Schmidt-Ruppin A-2) viral oncogene homolog (SRC) [87]. In addition, the impact of specific *KRAS* codon subtypes warrants further investigation in future clinical trials.

Trametinib is another selective and potent MEK inhibitor that has been clinically approved for *B-RAF* mutations, mainly to treat melanoma [81]. However, the drug showed similar PFS and a response rate as docetaxel in patients with previously treated *KRAS*-mutated NSCLC [88]. Activation of compensatory proteins/pathways may offer insight into why these clinical disappointments have occurred. A phase II trial with *KRAS*-driven NSCLC reported a trend for worse PFS in G12C cohorts compared to non-G12C patients (NCT02642042) [89].

#### 3.3.3. Other Downstream Inhibitors

KRAS oncogenic signaling also involves PI3K/AKT/mTOR via the interaction with the catalytic subunits of PI3K. Inhibiting *RAS*-driven PI3K activation blocks the progression of *KRAS^+^* tumors [90,91,92]. However, the mTOR inhibitor ridaforolimus as monotherapy displayed little clinical benefit in a phase II trial in *KRAS* mutant NSCLC resulting only in a modest increase in PFS [93]. Various phase I trials have been investigating the efficacy of targeting PI3K/AKT/mTOR pathway in combination with MEK inhibitors in patients with advanced-stage solid tumors including NSCLC [94,95,96]. Preliminary evidence of partial responses was reported in patients with solid tumors but not specifically in those with NSCLC. One potential obstacle for clinical approval of this approach might be that drug dosages for a complete abrogation of RAS signaling through PI3K/mTOR and MEK inhibition could be too high to be tolerated in humans [57].

The non-receptor tyrosine kinase focal adhesion kinase (FAK) involved in cell adhesion to the extracellular matrix is critical for cancer cell proliferation, survival, migration, and invasion and has also been explored as a target. The KRAS/RHOA/FAK pathway plays an important role in the development of lung tumors bearing inactivated *p53* or loss of *CDKN2A* [97]. A phase II trial of the FAK inhibitor defactinib failed to improve outcome in patients with *KRAS* mutant lung tumors [98] (NCT01951690). One reason behind might be co-occurring genetic events: FAK inhibitors have been documented to exert potent anti-tumor activity in NSCLC with *KRAS^G12V^* mutation in association with *CDKN2A* deficiency but not in *CDKN2A* wildtype background [97]. A phase Ib dose-finding, pharmacokinetic trial has been initiated to determine the maximum tolerated dose of the FAK inhibitor GSK2256098 and the MEK1/2 inhibitor trametinib in combination in eligible patients having mesothelioma or other solid tumors with MAPK activation [99]. Equivalent effect between GSK2256098 plus trametinib combination and those from an earlier GSK2256098 monotherapy study was observed in regard of reduction in FAK levels. The clinical efficacy of the combination was limited although safety profiles were acceptable up to the maximum tolerated dose.

The use of heat shock protein 90 (HSP90) inhibitors in *KRAS* mutant NSCLC also appeared to be a promising approach as these molecules target many KRAS downstream effectors. The HSP90 inhibitor AUY922 attenuated intrinsic resistance to PI3K and MEK inhibitors by suppressing both PI3K/AKT/mTOR and RAF/MEK/ERK signaling in *KRAS* mutant NSCLC cells [100,101]. Panaxynol is a natural HSP90 inhibitor and disrupts its function by binding to its N-terminal and C-terminal ATP-binding pockets. The compound inhibited the sphere forming ability of NSCLC cancer stem-like cells (CSCs) and suppressed the viability of NSCLC cells (non-CSCs) and their sublines with acquired chemoresistance, whereas normal cells derived from various organs were not affected [102]. Upon oral administration, panaxynol blocked lung tumorigenesis in *KRAS^G12D/+^* transgenic mice and mice carrying NSCLC xenografts without detectable toxicity. However, the clinical efficacy of HSP90 inhibitors is limited due to rapid resistance. In addition, in a phase III lung cancer trial of ganetespib (a HSP90 inhibitor) and docetaxel (an anti-microtubule agent), the combination failed to demonstrate any benefits. Hyperactivation of RAF/MEK/ERK/RSK and PI3K/AKT/mTOR pathways was defined as a key resistance mechanism to ganetespib [103]. Activity of p90 ribosomal S6 kinase (p90RSK; a key activator of the mTOR pathway and an ERK downstream target) was increased in *KRAS* mutant NSCLC ganetespib resistant cells. While genetic or pharmacologic inhibition of p90RSK restored sensitivity to ganetespib, reactivation of p90RSK and its downstream target M-phase inducer phosphatase 3 (CDC25C) promoted bypass of the ganetespib induced G_2_/M arrest and led to acquired resistance to ganetespib and cross-resistance to docetaxel in *KRAS* mutant NSCLC [103,104]. Simultaneous application of ganetespib and p90RSK or CDC25C inhibitors was highly synergistic, suggesting that the development of rationally designed HSP90 inhibitor combinations may prevent or overcome resistance to HSP90 inhibition [104].

## 4. New Optimism for Targeting *KRAS* Mutations

Advanced technologies in drug development and novel mechanistic insights into KRAS biology paved the way to refocus attention on strategies that directly interfere with the function of KRAS oncoproteins, with a particular interest in how to attack specific mutant alleles.

### 4.1. Covalent KRAS Inhibitors vs. KRAS Protein Degradation

One of the reasons for conducting indirect inhibition of *KRAS* was that in vivo blockage of the catalytic site with nucleotide competitive inhibitors is not feasible, at least in part due to the high affinity of RAS for GTP/GDP together with their high intracellular concentrations [105]. Recent advances have identified two classes of cysteine 12-modifying KRAS inhibitors which either bind to the nucleotide pocket of *KRAS* [106] or to the neighboring switch II region [107]. ARS-853, a potent G12C allele-specific inhibitor, reduced KRAS signaling and cancer cell growth in vitro [108,109]. These studies further illustrated that mutant RAS is not locked in a GTP-RAS constitutively active state: *KRAS*^G12C^ alteration enhances rapid nucleotide exchange (GDP–GTP) and thus leads to aberrant downstream signaling. This suggests that *KRAS* allelic substitutions are still sensitive to extracellular growth factors and their activity could be tackled by blocking upstream effectors. ARS-853 inactivated KRAS^G12C^ signaling by a trapping mechanism: *KRAS*^G12C^ was trapped in the GDP-RAS state, hindering further nucleotide exchange [108,109]. Concomitant treatment of ARS-853 with other tyrosine kinase inhibitors (TKIs) (e.g., EGFR TKI) or monoclonal antibodies blocking other receptor tyrosine kinases (RTKs) upstream of RAS showed synergistic activity [108,109]. Hence, complete blockage of RAS signaling may require combinatorial strategies with these compounds. However, ARS-853 has not entered clinical evaluations in *KRAS* mutant lung cancer as yet.

Optimization of the cysteine conjugation and the molecular interactions within the drug-induced pocket has led to the development of oral KRAS^G12C^ inhibitors: ARS-1620/ARS-3248 (Wellspring Biosciences), AMG 510 (Amgen/Carmot Therapeutics) and MRTX849 (Mirati Therapeutics) induced regression of *KRAS^G12C^* tumors [110,111,112]; the latter three entered phase I clinical trials (NCT04006301, NCT03600883, NCT03785249, NCT04303780, NCT04330664) [113]. Unfortunately, initial high hopes have been dampened by rapid adaptive resistance and MAPK signaling reactivation following inhibitor treatment [114,115].

Different from the covalent inhibitors that specifically target mutant *KRAS*, AZD4785 is a KRAS antisense-oligonucleotide (ASO) targeting *KRAS* gene irrespective of its mutational status [116]. AZD4785 potently depleted KRAS mRNA and protein, inhibited downstream effector pathways and led to anti-proliferative effects in mutant *KRAS* cells. Unlike observations with RAS/MAPK pathway inhibitors, ASO-driven depletion of *KRAS* was not associated with feedback activation of MAPK cascade. Anti-tumor activity was confirmed in mice harboring *KRAS*-mutated NSCLC xenografts and patient-derived xenografts. Despite the safety reports obtained in mice and monkeys, the first phase I trial in NSCLC and advanced solid tumors failed (NCT03101839). The underlying reason might be that AZD4785 does not distinguish between mutant and wildtype KRAS for degradation.

New optimism has been sparked by proteolysis-targeting chimeras (PROTACs) [80] targeting *KRAS*^G12C^ mutants. These bifunctional molecules simultaneously engage KRAS^G12C^ protein and an E3 ligase, forming a ternary complex, enabling the E3 ligase to ubiquitinate KRAS^G12C^ protein on proximal lysine residues. Zeng et al. has reported a cysteine 12-directed covalent degrader molecule incorporating ARS-1620 and thalidomide to recruit the cereblon E3 ligase which degraded an artificial GFP-KRAS^G12C^ fusion protein in reporter cells but not endogenous KRAS^G12C^ in pancreatic and lung cancer cells [117]. Recently, Bond et al. described the first endogenous KRAS^G12C^ degrader [118]. Docking of cysteine 12-directed covalent inhibitor MRTX849 into the switch II pocket of *KRAS^G12C^* revealed the pyrrolidine group to be solvent exposed; the linkers were extended directly from the pyrrolidine ring nitrogen. LC-2 was identified as the most potent KRAS^G12C^ degrading PROTAC, incorporating MRTX849 and the VHL E3 ligase ligand, which resulted in rapid engagement and sustained degradation. KRAS^G12C^ degradation by LC-2 depended on both proteasome and neddylation (neddylation of cullin 2, a VHL adaptor protein, is required for assembly and function of the VHL E3 ligase complex [119]) as pretreatment with epoxomicin or MLN4924, respectively, rescued KRAS^G12C^ levels. On the other hand, bafilomycin A1, an inhibitor of lysosomal acidification, proved ineffective indicating that LC-2-mediated KRAS^G12C^ degradation requires ternary complex formation with VHL and an intact ubiquitin proteasome system, whereas the lysosomal pathway is dispensable. Upon exposure to LC-2, phosphorylated ERK (pERK) signaling was attenuated for up to 72 h in multiple cancer cell lines including NSCLC cells. Total ERK was elevated in cells treated with the degrader molecule compared to vehicle controls at all time points indicating the initiation of a positive feedback loop upon KRAS^G12C^ degradation and pERK inhibition. As the covalent nature of LC-2 may limit its potency (because it cannot participate in multiple catalytic rounds of degradation), reversible degrader molecules attacking KRAS are needed. Ligand development for other *KRAS* mutations is ongoing. Although in vivo studies are awaited, the discovery of LC-2 is a milestone in anti-KRAS^G12C^ cancer therapy.

### 4.2. Improved Combinatorial Downstream Strategies for KRAS Mutant Lung Tumors

The clinical failure of agents against the RAF/MEK/ERK pathway downstream of RAS in *KRAS* mutant cancers has led to explore the possibility of using combinations of targeted agents inhibiting the multiple pathways downstream of RAS. Such a dual strategy provides the additional value of attacking two main downstream pathways and resulted in encouraging signals of activity in preclinical and in early phase trials.

Preclinical studies have suggested combinations of mTOR and MEK inhibitors in *KRAS* mutant lung cancers [120] but these attempts have proven challenging due to adverse events such as diarrhea, skin rash and fatigue [121]. Broutin et al. investigated the dependence of MEK and mTOR inhibition in a panel of *KRAS* mutant and *KRAS* wildtype NSCLC cell lines and showed for the first time that inhibition of mTOR but not MEK contributes to the majority of the growth inhibition in this combination [122]. There was no significant additive difference in growth inhibition caused by mTOR compared with mTOR plus MEK inhibition in *KRAS* wildtype cells, whereas the combination caused a significantly greater growth inhibition than either compound alone in *KRAS*-mutated cells. The results further suggest that in terms of toxicity, clinical trial designs using intermittent dosing should prioritize reducing the dose/frequency of the MEK inhibitor rather than mTOR inhibitor. Furthermore, Liang et al. recently reported that combinations of clinically approved mTOR inhibitors and chemotherapeutics synergized in inhibiting cell proliferation of *KRAS* mutant NSCLC cells in vitro and in vivo, and the efficacy of this approach correlated with the magnitude of mTOR activity induced by chemotherapy alone [123].

An open-label, phase Ib clinical trial has been initiated to determine the maximum tolerated dose and/or the recommended dose for use in a phase II trial for BKM120 (an oral PI3K inhibitor) in combination with binimetinib (an FDA-approved allosteric MEK inhibitor) (NCT01363232). This combination has been explored in patients with *EGFR* mutant pretreated NSCLC, triple-negative breast cancer, pancreatic cancer, colorectal cancer, malignant melanoma, NSCLC and other advanced solid tumors with *KRAS*, *NRAS* and/or *B-RAF* mutations; the results are not available yet. Another dose- and schedule-finding study evaluated MK-2206 (an allosteric inhibitor of pan-AKT) and selumetinib in patients with advanced treatment-refractory solid tumors (NCT01021748) [124]. Clinical anti-tumor activity was observed with durable tumor shrinkage in *KRAS*-mediated NSCLC and low-grade ovarian carcinoma. Strikingly, no responses were documented in *KRAS*-mutated colorectal or small-bowel carcinoma, partly due to distinct biological context differences in these diseases. Such heterogeneity of response among patients with *KRAS^+^* tumors mirrors the complexities of tumor biology and the presence of other aberrant driver mutations or disruption of signaling feedback loops.

A recent study reported that MEK/bromodomain and extraterminal domain (BET) inhibitors suppress pre-replication complex proteins in cycling cells and trigger stalled replication, DNA damage and death [125]. This combination is effective in half of *KRAS*-mutated NSCLC and cause potent tumor regression in xenograft and PDX models. Another potential combinatorial downstream strategy for *KRAS*-mutated lung tumors employed a short hairpin RNA screen [126]. Manchado et al. reported that the MEK inhibitor trametinib provokes a compensatory response via *FGFR-1*-mediated adaptive resistance in *KRAS* mutant lung cancer cells. As a consequence, genetic or chemical blockage of FGFR-1 (with ponatinib) together with trametinib enhances cell death in *KRAS^G12V^* positive tumors in vitro and in vivo. Recently, Molina-Arcas et al. described a promising combinatorial strategy using mTOR, insulin-like growth factor 1 receptor (IGF1R) and MEK inhibitors to treat lung tumors bearing *KRAS* allelic substitutions [127]. Marked tumor regression was achieved in various mouse models. While each component of this triple drug combination has been found to cause adverse events in clinical trials, substitution of MEK inhibitor with the recently developed allele-specific KRAS^G12C^ inhibitor ARS-1620 allowed strong inhibition of *KRAS^G12C^* positive tumors with low toxicity and markedly boosted the effects of ARS-1620 monotherapy. This study provides insights into how best to use KRAS^G12C^ specific inhibitors in the clinic. However, one notion to be aware of is that cell line-based and mouse models may prove challenging to extrapolate to future clinical studies. Possible toxicities upon combinations of targeted agents are often not easy to predict and can be difficult to identify in preclinical studies. The triple therapeutic approach described above aims to prevent growth signaling pathway reactivation by crosstalk and negative feedback, yet there is no guarantee that it will not be overridden by acquisition of drug resistance due to pre-existing tumor heterogeneity in patients [127]. Therefore, it is likely that other therapeutic paths including immunotherapy, radiotherapy and chemotherapy would need to be integrated.

Further exciting preliminary activity with low frequency of adverse events has been documented for a potent RAF/MEK inhibitor (RO5126766). Patients with *KRAS* mutant lung adenocarcinomas showed significant treatment benefits, with 60% of patients showing tumor reductions—of which, 30% were partial responses [128]. The significant response rate represents one of the first responses to single agent downstream inhibitors to date. A multi-center, dose-escalation, phase I trial is ongoing to assess the maximum tolerated dose and the pharmacodynamic activity of the combination RO5126766 plus the FAK inhibitor defactinib in advanced solid tumors including *KRAS^+^* NSCLC (NCT03875820). Both compounds will be administered orally twice a week for 3 weeks followed by 1 week off, RO5126766 under fasting conditions and defactinib immediately after a meal. A second study aims to test the safety of RO5126766 at different doses for patients with advanced *KRAS^+^* lung cancer who have previously received treatment with a programmed cell death protein 1 (PD-1) or programmed cell death 1 ligand 1 (PD-L1) inhibitor (NCT03681483).

Recent functional studies demonstrated that the switch region of RAS interacts with a large number of effector proteins with a conserved RAS-binding domain (RBD) [129]. Because RBD-mediated interactions are essential for RAS signaling, blocking RBD association with small molecules offers a promising route to success. One such compound called rigosertib acts as a RAS mimetic and targets RBDs across multiple signaling pathways involving RAF kinases, RALGDS and PI3Ks [129]. This interaction hampers their inability to bind to RAS and subsequently abrogates downstream signaling. The therapeutic efficacy of rigosertib is currently being evaluated in several clinical trials across different types of cancer. A phase I/IIa trial is designed to study the combination of rigosertib plus nivolumab (an immune checkpoint inhibitor) in metastatic *KRAS^+^* lung adenocarcinoma adult patients who have progressed on standard first-line treatment (NCT04263090). Rigosertib will be dosed twice a day for 21 consecutive days, followed by 7 days off treatment, while nivolumab will be administered twice per 28 day cycle.

### 4.3. Second Chance for EGFR-related Signaling in KRAS-driven NSCLC as Therapeutic Benefit

In wildtype cases, KRAS is activated in response to ligand-activated signaling through ERBB RTKs (Figure 1) [130]. Strikingly, initial clinical trials using first-generation EGFR TKIs erlotinib or gefitinib have demonstrated little or no benefit to patients with *KRAS* mutations and the presence of mutant *KRAS* is used as a biomarker to exclude patients for EGFR TKI therapy [75,131,132,133]. Two meta-analyses suggested that patients with *KRAS*-mutated tumors exhibited significantly lower response rates with EGFR TKI than those with *KRAS* wildtype tumors [134,135]. However, in both studies, the *KRAS* wildtype group harbored *EGFR* alterations, hindering interpretation of the results. The reason behind the negative treatment interaction between EGFR TKI and *KRAS* mutant tumors was believed to be that oncogenic mutations in *KRAS* lock the protein in a constitutively active state, conferring independence from upstream signaling by ERBB RTKs (Figure 1). However, recent data provide evidence that this independence may not be definite: (i) in *KRAS* mutant NSCLC cell lines, activation of PI3K depends on basal activity of wildtype IGF1R, illustrating a model for how oncogenic and normal signal transduction coordinate [136]; (ii) development of *KRAS*^G12D^-driven pancreatic ductal adenocarcinoma is suppressed in the absence of *EGFR* [137,138]; and (iii) induced expression of ERBB2 and ERBB3 provokes resistance to MEK inhibition in *KRAS^+^* lung and colorectal cell lines [139].

Two preclinical studies further challenge this dogma, indicating that mutant *KRAS* demands activation of ERBB receptors to facilitate lung tumorigenesis [140,141]. Multiple ERBB RTKs and several of their ligands are expressed and active from the earliest stage of *KRAS*-driven tumor development in men and mice [140,141]. Deletion of *EGFR* in a mouse model of autochthonous lung cancer driven by *KRAS^G12D^* reduced KRAS activity and transiently retarded tumor growth [140]. However, a rapid resistance mechanism by non-EGFR ERBB family members was observed at late tumor stages. This escape mechanism explains the disappointing outcome of first-generation TKIs and suggests high therapeutic potential of pan-ERBB inhibitors. In fact, the FDA-approved pan-ERBB inhibitor afatinib (as monotherapy) improved overall survival as shown in genetically engineered mouse models and in patient- and cell line-derived xenografts [140], whereas neratinib (another pan-ERBB inhibitor) proved to be highly efficient only in combination with MEK inhibition in an autochthonous tumor setting [141]. This difference in application (single agent vs. combinatorial) may stem from different dosing schedules and/or mouse models used. Taken together, these studies provide a solid ground to re-evaluate the use of pan-ERBB inhibitors in clinical trials in patients with *KRAS* mutant lung tumors.

### 4.4. Metabolic Rewiring and Autophagy Inhibition

The reprogramming of cellular metabolism is an important hallmark of cancer typically associated with increased growth demands, and/or compensatory adaptation to mitochondrial defects [142,143,144]. Mutant KRAS activity causes increased proliferation in multiple models. Hence, it is not surprising that high-grade *KRAS^+^* lung tumors rely on accelerated glucose metabolism for enhanced antioxidant potential and exhibit increased autophagy and macropinocytosis, relative to non-mutant cells [145,146,147]. In line, advanced-stage tumors in *KRAS^G12D/+^/p53^-/-^* mice were hypersensitive to combined glucose and glutathione depletion, whereas low-grade tumors in the same model, with a glucose metabolism similar to that of normal tissues, failed to respond [33]. Unfortunately, glucose depletion may pose significant toxicity risks in the clinics. An alternative strategy may be targeting of tumor-specific phenotypes associated with the metabolic rewiring. Multiple glycolytic genes are upregulated in NSCLC—some of which correlate with poor prognosis [148,149,150,151,152]. Deletion of *hexokinase 2 (Hk2)* in lung cancer cells suppressed glucose derived ribonucleotides and impaired glutamine derived carbon utilization in anaplerosis [153]. Its systemic ablation was found to be therapeutic in *KRAS^G12D/+^* mice bearing lung tumors without adverse physiological consequences. In addition, silencing of *pyruvate kinase isoenzyme M2* enhanced the efficacy of docetaxel and cisplatin (an alkylating agent) in human *KRAS* mutant lung cancer xenograft models [154,155]. Ablation of *lactate dehdryogenase-a enzyme* (critical for interconversion of pyruvate to lactate and associated with aggressive outcome) repressed the formation of spontaneous *KRAS^G12D^*-driven lung tumors [156].

Autophagy is a catabolic pathway in cells to support metabolism in response to starvation and to clear damaged proteins and organelles. It has also been identified as a survival mechanism across several cancer types [157,158,159,160]. The association between tumor cell survival and autophagy can be explained, at least partly by the role of autophagy in protecting cells from undergoing programmed cell death [161]. This provides a logical rationale for why the inhibition of autophagy could improve the response to other agents and forms the basis for completed and ongoing clinical trials for NSCLC (NCT01649947, NCT00728845, NCT01497925, and NCT03057340). In fact, in recent years, inhibition of autophagy has been widely explored as a potential therapeutic intervention for cancer, although the results are somehow contradictory. Tumors with *KRAS* substitutions were reported to depend on macroautophagy to cope with oncogene-induced metabolic stress in order to facilitate the maintenance of mitochondrial metabolism, cell survival and tumorigenic growth [162,163]. This paradigm has been questioned by the findings of Eng et al. [164]: deletion of *autophagy-related 7* potently blocked macroautophagy in several cancer lines with oncogenic *KRAS* alterations but failed to inhibit cell proliferation in vitro or tumorigenesis in vivo. On the other hand, it has been shown that inhibition of RAF/MEK/ERK cascade activates serine/threonine kinase 11/AMP-activated protein kinase/Unc-51-like autophagy activating kinase 1 (STK11/AMPK/ULK1) signaling axis, leading to autophagy induction [165,166,167]. This process of cellular recycling protects tumor cells from cytotoxic effects of KRAS pathway blockage. Of note, it is likely that other pathways are also involved in autophagy induction in response to inhibition of RAF/MEK/ERK signaling. Three studies recently published simultaneously illustrated synergistic activity of concomitant inhibition of autophagy and MAPK pathway in patient-derived xenografts of *KRAS* mutant pancreatic ductal adenocarcinoma, *NRAS* mutant melanoma and *B-RAF* mutant colorectal tumor [165,166,167]. Of clinical relevance, off-label treatment of a patient with metastatic pancreatic ductal adenocarcinoma with the MEK inhibitor trametinib plus hydroxychloroquine (a lysosomotropic agent for autophagy blockage) resulted in 50% shrinkage in tumor burden without any adverse effects [166]. In line, a phase I trial is recruiting patients with *KRAS^+^* metastatic pancreatic cancer to study the best dose of hydroxychloroquine when given together with the MEK1/2 inhibitor binimetinib (NCT04132505). Based on these intriguing results, future investigations are required to address the potential therapeutic benefit of combined inhibition of autophagy and MAPK signaling for patients with *KRAS*-mutated NSCLC.

In light of the role of autophagy inhibition on mitochondrial functionality and tumor maintenance [162,168], direct targeting of mitochondrial activity may also be of therapeutic relevance. Disruption of mitochondrial function by loss of *mitochondrial transcription factor A* hampered *KRAS^G12D^*^/+^ lung tumor formation in mice [169]. Phenformin monotherapy, a mitochondrial complex I inhibitor, has been defined as a selective agent targeting lung tumors in *KRAS^G12D^*^/+^/*STK11*^-/-^ mice; no changes in tumor burden and survival have been observed in *KRAS^G12D^*^/+^/*p53*^-/-^ context [170]. Mutant *KRAS* tumors also express unique dependencies in terms of fatty acid synthesis and oxidation, tricarboxylic acid cycle (TCA)-dependent glucose metabolism and branched-chain amino acid utilization, which can potentially be exploited therapeutically [145]. However, their metabolic heterogeneity may severely limit the efficacy. Future studies thus are needed to address this therapeutic challenge.

Metabolic communication in tumors also adds a new layer of immunoregulation for immune evasion [171] and its targeting is an encouraging strategy to enhance tumor immunogenicity [172]. Altered metabolic programs affect the fate decision of T cells [173]. However, tumors with distinct metabolic properties may modulate T cell responses differently, enhancing the chance of resistance to treatment [145,171,172,174]. Similarly, agents that target tumor cell metabolism may yield unexpected results due to their potential effects on stroma cells and stroma-tumor cell communication. Future in vivo models will offer invaluable insight onto the evaluation of stromal effects on metabolic targeting and resistance to therapy in *KRAS^+^* NSCLC.

### 4.5. Immunotherapy

Cancers escape immune surveillance by aberrantly expressing immune checkpoints (e.g., upregulation of the immunosuppressive protein PD-L1) that masks them from the host immune system. Immune checkpoint blockage using monoclonal antibodies against PD-1 and its main ligand PD-L1 has substantially improved the treatment landscape of advanced-stage NSCLC and made its most strong impact in the stage III and first-line stage IV settings [175,176,177,178]. This treatment is being progressively incorporated into the first-line armamentarium [179] (NCT02259621, NCT03693326); however, response rates reach only 20% in unselected patients [180].

The key limitation of the advances in immunotherapy has been the identification of a biomarker for a sensitive and specific prediction of treatment response. Lung cancer patients with T cell inflamed tumor phenotype and high tumor mutational burden obtain greatest benefits upon exposure to immune checkpoint inhibitors [57,71,181]. PD-L1 expression is the only FDA-approved biomarker for these therapies in patients with lung adenocarcinoma but its sensitivity is modest. Studies on the predictive importance of *KRAS* mutations for the efficacy of immune checkpoint inhibitors have not uniformly provided positive results. Mutant *KRAS* was associated with increases in tumor infiltrating lymphocytes, PD-L1 expression and tumor mutational burden [182,183]. However, while Gianoncelli et al. observed no significant differences between *KRAS^+^* and non-*KRAS* NSCLC patients in terms of progression-free or overall survival [184], Kauffmann-Guerrero et al. reported a positive outcome for *KRAS* mutations in response to immune checkpoint inhibitors [185]. Molecular diversity within *KRAS^+^* NSCLC patients offers an attractive biological explanation for such discrepancy in results [186]. In particular, co-alterations in *STK11* and *p53* (both associated with high tumor mutational burden) profoundly influence the tumor-immune contexture [21]. *KRAS/STK11* NSCLC (25% of *KRAS^+^* NSCLC) lacked tumor-infiltrating lymphocytes and expressed reduced levels of immune markers and PD-L1. Inactivating somatic mutations in *STK11* have thus emerged as a major genomic driver of primary resistance to immune checkpoint inhibitors [187]. Such negative impact extends to tumors with high PD-L1 expression and is further associated with primary resistance to combined PD-L-1/cytotoxic T-lymphocyte associated-protein 4 (CTLA-4) blockage in NSCLC [188]. Simultaneous therapies that reverse the T cell suppressive tumor microenvironment in *KRAS/STK11* NSCLC would be required for a potent response. In fact, recent data demonstrated how anti-PD-1 antibodies failed to induce tumor regression in mice without *STK11* but exposing them to an interleukin-6 antibody (a pro-inflammatory cytokine secreted by tumor-infiltrating neutrophils) or neutrophil-depleting antibody yielded T cell infiltration and therapeutic benefits [189]. Similarly, anti-angiogenics and epigenetic modifiers are also needed to be investigated in *KRAS/STK11* NSCLC. In addition, mutations in *phosphatase and tensin homolog (PTEN)* and *KEAP1* have been nominated as potential drivers of de novo resistance to immune checkpoint inhibition in NSCLC [188,190]. Co-mutation in *KEAP1* was also associated with shorter duration of initial platinum-based chemotherapy and shorter overall survival from start of immune therapy in *KRAS^+^* advanced NSCLC [190]. In contrast, *KRAS/p53* co-mutated NSCLC was characterized by an inflammatory response, immune-editing and expression of co-stimulatory and co-inhibitor molecules including PD-L1 [187,191]. These observations indicate that this cohort may be particularly susceptible to anti-immune checkpoint therapies. Indeed, *KRAS/p53* co-mutations were associated with potent treatment response and improved progression-free and overall survival [187]. Taken together, co-occurring somatic genomic alterations in *KRAS^+^* NSCLC represent independent predictors for sensitivity to anti-PD-1/PD-L1 therapies. Treatment needs to be individualized and may require the use of rational combinations (e.g., immunotherapy plus conventional therapies targeting RAS downstream cascade or cell cycle inhibitors) for a durable therapy response in *KRAS^+^* NSCLC [57]. In immune-competent mice, the KRAS^G12C^ inhibitor AMG 510 resulted in a pro-inflammatory tumor microenvironment and produced durable cures in combination with immune checkpoint inhibitors [111]. Several clinical trials are ongoing. A phase I study evaluates the combination of the CDK4/6 inhibitor abemaciclib with anti-PD-1 antibody pembrolizumab for *KRAS* mutant, *PD-L1^+^* patients with stage IV NSCLC (NCT02779751). A second trial is recruiting patients to determine the side effects and best dose of pembrolizumab and the MEK inhibitor trametinib for recurrent *KRAS*-mutated NSCLC that is metastatic, unresectable or locally advanced (NCT03299088, NCT03225664). Another phase I study focuses on the safety and tolerability of pembrolizumab infusion in combination with mRNA-5671/V941 (a KRAS vaccine) in patients with *KRAS* mutant advanced or metastatic NSCLC (NCT03948763). An open-label phase II study aims to assess the safety and efficacy of SHR-1210 (an anti-PD-1 antibody) plus apatinib mesylate (an anti-vascular endothelial growth factor (VEGF) receptor-2 inhibitor) versus pemetrexed (an antimetabolite) and carboplatin (an alkylating agent) in adult patients with *KRAS*^+^ stage IV NSCLC (NCT03777124). Another phase II trial is underway to determine the effects of combining pembrolizumab with anti-VEGF receptor (ramucirumab), and docetaxel in treating NSCLC patients who failed to respond to FDA-approved treatments with platinum-based chemotherapy administered concurrently or sequentially with anti-PD-1/PD-L1 immunotherapy (NCT04340882).

It is also notable that responsiveness to PD-1/PD-L1 checkpoint blockage correlates with an interferon gamma-inducible gene signature and major histocompatibility complex class II (MHC II) expression by tumor cells [192]. Recent data emphasize the active inhibitory role of epigenetic and ERK signaling cascades in restricting cancer cell-intrinsic MHC II expression in NSCLC and suggest their combinatorial inhibition as new responsiveness to checkpoint therapies [192].

Based on the limitations of both immune and targeted therapies a recent study aimed to combine these two modalities to treat *KRAS* mutant lung tumors [193]. As opposed to conventional continuous exposure to MEK inhibitors in the clinic, a pulsatile schedule of MEK inhibitors (selumetinib and trametinib) was more effective at controlling tumor progression and altering the tumor microenvironment favorably by enhancing T cell activation with increased expression of immune checkpoint regulators CTLA-4 and PD-1. Other MEK inhibitors with different potency, target specificity and elimination half-life such as cobimetinib and binimetinib also need to be tested. In vivo data with pulsatile MEK inhibition in a transplantable *KRAS* model and in a genetically engineered mouse model bearing human *KRAS^G12C^* substitution were consistent with the ex vivo observations: the pulsatile treatment had a superior anti-tumor activity and delayed drug resistance in comparison with continuous exposure. This effect was further enhanced when combined with CTLA-4 blockage. Such prolonged survival was not present in immune-deficient mice illustrating that it was mediated by the adaptive immune system. These results set the foundation and highlight the importance of a combinatorial therapeutic strategy using pulsatile targeted therapy together with immunotherapy to optimally enhance tumor delay and promote long-term anti-tumor immunity. A similar pulsatile treatment regimen is under clinical evaluation in NSCLC patients with intermittent selumetinib and antibodies targeting CTLA-4 and PD-L1. One drawback, however, is that the trial did not stratify for co-existing alterations apart from *KRAS*. Hence, it is likely that MEK inhibition alone, which targets downstream of KRAS, may not suffice to repress *KRAS*-driven tumors together with additional alterations, even when combined with immunotherapy.

In addition, Tran et al. reported that infusion of CD8^+^ T cells targeting *KRAS^G12D^* results in regression of lung metastases and thus mediates effective anti-tumor activity in a patient with metastatic colorectal cancer [194]. This immune response is restricted to the presence of the major histocompatibility complex class I allele HLA-C*08:02: the authors observed progression of one of the lesions when the chromosome 6 haplotype encoding HLA-C*08:02 was lost. Despite the rarity of HLA-C*08:02, the study illustrated the promise of T cell-based immunotherapy for targeting *KRAS^G12D^* and HLA-C*08:02. Further evaluation in more patients and testing for other *KRAS* allelic substitutions is needed. A novel clinical trial is recruiting patients to determine the safety of administering peripheral blood lymphocytes transduced with anti-KRAS^G12D^ murine T cell receptor (TCR) with preparative lymphodepletion and high dose interleukin-2 (NCT03745326). Its phase II part will focus on whether solid tumors (gastrointestinal, pancreatic, gastric, colon and rectal) will shrink upon treatment. Another trial (NCT04146298) will investigate the safety and activity of adoptive transfer of autologous T cells genetically engineered to express a TCR that targets *KRAS^G12V^* in the context of HLA-A*11:01 in human leukocyte antigen (LA)-matched patients with advanced pancreatic cancer; the study will also measure the in vivo survival of engineered T cells. Future studies involving anti-mutant KRAS TCR in NSCLC are awaited.

## 5. Discussion

Despite decades of research highlighting mutant *KRAS* as a central driver of tumorigenesis and clinical resistance, the development of therapeutics potently tackling *KRAS* aberrations has so far been unaccomplished. In recent years, a variety of efficient and specific chemicals has entered preclinical and early clinical settings. A new wave of attempts is motivated to drug *KRAS* directly, which has long been considered difficult to target. A striking breakthrough has been achieved with covalent inhibitors such as MRTX849 and AMG 510 as well as with LC-2, a degrader molecule against the endogenous protein, in patients with *KRAS^G12C^* lung tumors. Conventional (e.g., combined chemotherapy with targeted KRAS downstream agents) and novel tools based on improved understanding of the pathobiology of mutant *KRAS* (e.g., cellular co-dependencies, association with a vulnerability to immunotherapies) are appealing strategies but demand further clinical testing in patients. Rational combinations may further advance the attempts to target *KRAS*-driven lung tumors. The reason behind the insufficient benefits of treating *KRAS^+^* NSCLC patients might be mechanism-related rather than the potency and efficacy of targeting itself. A critical point is the high heterogeneity within *KRAS* mutant lung tumors. Co-existing genetic events and mutant *KRAS* allele copy number gains define distinct metabolic profiles and tumor microenvironment, both contributing to differential drug sensitivities in seemingly comparable tumors. Thus, by molecularly tailored stratification in addition to *KRAS* aberrations we may finally change the course of the deadly NSCLC.

## Abbreviations

A-RAF	V-Raf murine sarcoma 3611 viral oncogene homolog 1
AKT	Protein kinase B
ALK	Anaplastic lymphoma receptor tyrosine kinase
AMPK	AMP-activated protein kinase
APC/C	Anaphase-promoting complex/cyclosome
ASK1	Apoptosis signal-regulating kinase 1
ASO	Antisense-oligonucleotide
B-RAF	V-Raf murine sarcoma viral oncogene homolog B
Bad	BCL2-associated agonist of cell death
BCL-XL	B-cell lymphoma-extra large
BCL2	B-cell lymphoma 2
BET	Bromodomain and extraterminal domain
BRCA	Breast cancer gene
C-RAF	V-Raf-1 murine leukemia viral oncogene-like protein 1
CDC25C	M-phase inducer phosphatase 3
CDK	Cyclin-dependent kinase
CDKN2A/B	Cyclin-dependent kinase inhibitor 2A/B
Chk1	Checkpoint kinase 1
CRISPR/Cas9	Clustered regularly interspaced short palindromic repeats/CRISPR-associated protein 9
CSC	Cancer stem-like cell
CTLA-4	Cytotoxic T-lymphocyte-associated protein 4
DDR1	Discoidin domain receptor 1
EGFR	Epidermal growth factor receptor
ERK	Extracellular regulated kinase
FAK	Focal adhesion kinase
FDA	U.S. Food and Drug Administration
FGFR	Fibroblast growth factor receptor
FTI	Farnesyltransferase inhibitor
G12C	Glycine 12 to cysteine
G12D	Glycine 12 to aspartic acid
G12V	Glycine 12 to valine
GAP	GTPase-activating protein
GATA2	GATA-binding protein 2
GDP	Guanosine diphosphate
GEF	Guanine nucleotide exchange factor
GGTase I	Geranylgeranyltransferase I
GTP	Guanosine triphosphate
HER2–4/ERBB2–4	Human epidermal growth factor receptors 2–4
HLA	Human leukocyte antigen
HRAS	Harvey rat sarcoma viral oncogene homolog
HSP90	Heat shock protein 90
ICMT1	Isoprenyl carboxyl methyltransferase
IGF1R	Insulin-like growth factor 1 receptor
IKK	IkappaB kinase
IκB	Nuclear factor of kappa light polypeptide gene enhancer in B-cells inhibitor
JNK	c-Jun N-terminal kinase
KEAP1	Kelch-like ECH-associated protein 1
KRAS	Kirsten rat sarcoma viral oncogene homolog
LKB1	Liver kinase B1
MAP3K7	Mitogen-activated protein kinase kinase kinase 7
MAPK	Mitogen-activated protein kinase
MEK	Mitogen-activated protein kinase kinase
MHC II	Major histocompatibility complex class II
mTOR	Mechanistic target of rapamycin kinase
NF-κB	Nuclear factor ‘kappa-light-chain-enhancer’ of activated B-cells
NRAS	Neuroblastoma rat sarcoma viral oncogene homolog
NSCLC	Non-small-cell lung cancer
ORR	Objective response rate
p53	Tumor protein p53
p90RSK	p90 ribosomal S6 kinase
PARP	Poly(ADP-ribose)polymerase
PD-1	Programmed cell death protein 1
PD-L1	Programmed cell death 1 ligand 1
PDX	Patient-derived xenograft
pERK	Phosphorylated ERK
PFS	Progression-free survival
PI3K	Phosphoinositide 3-kinase
PLD1	Phospholipase D1
PLK1	Polo-like kinase 1
PROTAC	Proteolysis-targeting chimera
PTEN	Phosphatase and tensin homolog
PTPN11	Protein tyrosine phosphatase non-receptor type 11
RAC	Ras-related C3 botulinum toxin substrate 1
RAF	Rat fibrosarcoma
RAL	Ras-like protein
RALGDS	Ral guanine nucleotide dissociation stimulator
RBD	RAS-binding domain
RCE1	RAS-converting enzyme 1
RET	RET proto-oncogene
RHOA	Ras homolog family member
RNAi	RNA interference
ROCK2	Rho-associated coiled-coil containing protein kinase 2
ROS	Proto-oncogene tyrosine-protein kinase ROS
RTK	Receptor tyrosine kinase
SNAIL2	Snail family transcriptional repressor 2
SRC	V-Src avian sarcoma (Schmidt-Ruppin A-2) viral oncogene homolog
STK11	Serine/threonine kinase 11
STK3	Serine/threonine-protein kinase 3
TBK1	TANK binding kinase 1
TCA	Tricarboxylic acid cycle
TCR	T cell receptor
Th17	T helper 17
TIAM1	T lymphoma invasion and metastasis-inducing protein 1
Tim-3	T cell immunoglobulin mucin-3
TKI	Tyrosine kinase inhibitor
TPX2	Targeting protein for Xklp2
Treg	Regulatory T cells
ULK1	Unc-51-like autophagy activating kinase 1
VHL	von Hippel–Lindau disease tumor suppressor
WT1	Wilms tumor 1
